# C-PAmP: Large Scale Analysis and Database Construction Containing High Scoring Computationally Predicted Antimicrobial Peptides for All the Available Plant Species

**DOI:** 10.1371/journal.pone.0079728

**Published:** 2013-11-11

**Authors:** Anastasia Niarchou, Anastasia Alexandridou, Emmanouil Athanasiadis, George Spyrou

**Affiliations:** Biomedical Research Foundation of the Academy of Athens, Athens, Greece; University of Malaya, Malaysia

## Abstract

**Background:**

Antimicrobial peptides are a promising alternative to conventional antibiotics. Plants are an important source of such peptides; their pharmacological properties are known since antiquity. Access to relevant information, however, is not straightforward, as there are practically no major repositories of experimentally validated and/or predicted plant antimicrobial peptides. PhytAMP is the only database dedicated to plant peptides with confirmed antimicrobial action, holding 273 entries. Data on such peptides can be otherwise retrieved from generic repositories.

**Description:**

We present C-PAmP, a database of computationally predicted plant antimicrobial peptides. C-PAmP contains 15,174,905 peptides, 5–100 amino acids long, derived from 33,877 proteins of 2,112 plant species in UniProtKB/Swiss-Prot. Its web interface allows queries based on peptide/protein sequence, protein accession number and species. Users can view the corresponding predicted peptides along with their probability score, their classification according to the Collection of Anti-Microbial Peptides (CAMP), and their PhytAMP id where applicable. Moreover, users can visualise protein regions with a high concentration of predicted antimicrobial peptides. In order to identify potential antimicrobial peptides we used a classification algorithm, based on a modified version of the pseudo amino acid concept. The classifier tested all subsequences ranging from 5 to 100 amino acids of the plant proteins in UniProtKB/Swiss-Prot and stored those classified as antimicrobial with a high probability score (>90%). Its performance measures across a 10-fold cross-validation are more than satisfactory (accuracy: 0.91, sensitivity: 0.93, specificity: 0.90) and it succeeded in classifying 99.5% of the PhytAMP peptides correctly.

**Conclusions:**

We have compiled a major repository of predicted plant antimicrobial peptides using a highly performing classification algorithm. Our repository is accessible from the web and supports multiple querying options to optimise data retrieval. We hope it will greatly benefit drug design research by significantly limiting the range of plant peptides to be experimentally tested for antimicrobial activity.

## Background

Antimicrobial peptides constitute a crucial part of plant defence against pathogens [1]. Such peptides have been extracted from leaves, flowers, stems, seeds and roots and can be broadly grouped into three categories, namely thionins [2], defensins [3] and lipid transfer proteins [4]. Thionins are small, positively charged peptides, enriched in arginine, lysine, and cysteine and act against fungi, bacteria and animal and plant cells. Defensins, originally classified as a type of thionins, are also abundant in basic residues. They exhibit strong antifungal action, but their role in vivo is otherwise still poorly understood. In vitro, they are known to inhibit translation in both mammalian and non-mammalian cells. The defensive properties of lipid transfer proteins have not been fully elucidated yet, but they have been observed to act against fungi and bacteria.

Availability of information on plant antimicrobial peptides remains limited, despite their established role in combating pathogens and potential uses in medicine and agriculture. There is only one central repository of experimentally validated plant antimicrobial peptides, PhytAMP, with 273 entries [5]. Otherwise, plant peptides can be found in databases [6]–[9] containing antimicrobial peptides from various species. Of these databases, CAMP is the only one to hold predicted peptides as well [10].

We constructed a database of predicted antimicrobial peptides from plants, which we hope will complement PhytAMP and promote research in antimicrobial compound design. Potential antimicrobial candidates were selected by a machine learning algorithm and were stored along with relevant information in a database, which is easily accessible from the web.

## Construction and Content

### Datasets

Our positive dataset consists of a set of 2,160 experimentally validated antimicrobial peptides found in the antimicrobial peptide database (APD) [11] and CAMP. This set was obtained by filtering the APD and CAMP peptides through CD-HIT [12] to eliminate sequences at >85% identity.

Our negative dataset consists of three types of sequences: random subsequences of UniProt/SwissProt proteins that have not been described or annotated as antimicrobial, synthesized amino acid sequences following a uniform amino acid distribution and synthesized amino acid sequences following the amino acid distribution of UniProt/SwissProt. The former type of sequences was selected using BioPython [13] whereas the latter two types of sequences were generated using GenRGenS [14]. We used a diverse training set to avoid overfitting the classifier. The initial negative dataset consisted of 4,000 sequences (2,000 random protein fragments and 2,000 artificial sequences, 1,000 of each type), 3,983 of which remained after applying CD-HIT to eliminate sequences at >85% identity. All sequences in the negative dataset are less than150 amino acids long.

### Feature Selection

The feature vector consists of the pseudo-amino-acid composition [15] with respect to the E1 amino acid descriptor as described in [16]. In this paper, 237 physicochemical descriptors of amino acids are transformed to a set of 5 quantitative descriptors that allow amino acids to maintain roughly the same distribution as in the original 237-dimensional property space. Specifically, the 5 descriptors are the eigenvectors corresponding to the first 5 eigenvalues of the matrix containing the scalar products between all pairs of the original 237-dimensional vectors. Among these descriptors (termed E1–E5), the E1 descriptor was found to be highly correlated with hydrophobicity/hydrophilicity, polarity and charge, all of which have been associated with antimicrobial action.

Moreover, the pseudo-amino-acid composition provides information on both amino acid composition and the relative positioning of amino acids within the sequence. Within this framework an amino acid sequence is represented by a 20+*λ* vector. The first 20 dimensions correspond to amino acid composition and the subsequent *λ* dimensions represent sequence order correlations from all the most contiguous residues of a sequence to all *λ* most contiguous ones. For instance, if *λ* equals 2, we would have a 22-dimensional vector whose 21^st^ and 22^nd^ dimensions would reflect correlations among all the most contiguous and all the second most contiguous amino acids respectively. The feature vector is given by (1):



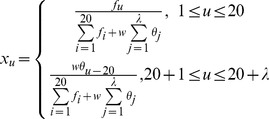
(1)where *f_u_* is the normalised occurrence frequency of the 20 amino acids, *w* is the weight of the amino acid ordering effect and *θ_j_* is the j-th tier correlation, which in our case is:



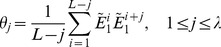
(2)where *L* is the sequence length and 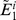 is the normalized E1 value for the amino acid at position *i*:
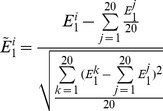
(3)


We set *λ* equal to 4 (our shortest peptide is 5 amino acids long), resulting to 24-dimensional feature vectors.

Since the E1 amino acid descriptor comprises several physical properties related to antimicrobial activity and the pseudo-amino-acid composition formulation allows us to use structural information as well, their combination is optimally suited to our purposes. It should be noted that this method will identify sequences of similar physical and, therefore, functional properties even if they are highly dissimilar. Besides, the pseudo-amino-acid composition has already been successfully used in predicting functional properties of proteins and peptides [15].

### Classification

The classifier used in the present study is a Support Vector Machine (SVM) as implemented in the scikit-learn package [17]. SVMs have been successfully used in protein functional classification and localisation problems in the past (e.g. [18], [19], [20]), and in the context of antimicrobial activity prediction in particular (Table 1). The implementation is based on LIBSVM [21], which also outputs probability values for each class. Probabilities are computed using an improved version of Platt's suggestion [22]:
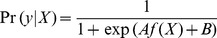
(4)where Pr(y|X) is the posterior probability that point X belongs to class y and f(X) is the signed distance of X from the separating hyperplane. A and B are obtained by minimizing the likelihood function. LIBSVM uses a solver that ensures global convergence and uses cross-validation to avoid overfitting [23], [24].

**Table 1:** Comparative overview of other antimicrobial peptide studies.

Study	Method	Accuracy	Features	Positive set	Negative set	Validation set
CAMP	SVMRandom Forests	91.50%93.2%	64 (after recursive feature elimination on initial set of 257) physicochemical properties (composition), dipeptide & tripeptide frequencies, distribution & transition of some features along sequences	2578 experimentally validated CAMP peptides	4011 random proteins from UniProt, synthesized sequences using random numbers, experimentally verified non-antimicrobial peptides (25)	30% of positive & negative sets
Fjell et al	Quantitative structure-activity relationships (QSAR)	80.00%	44 QSAR descriptors	1433 synthesized peptides, 9 amino-acids long(antibacterial acitivity measured experimentally)		∼100000 synthesized peptides
Torrent et al	ANNSVM	90%75%	8 physicochemical & structural properties (50 hidden neurons)	1157 CAMP antimicrobial peptides	991 randomly selected UniProt protein fragments	290 antimicrobial peptides from CAMEL and RANDOM databases
Porto et al	SVM	83.02%	4 physicochemical properties	199 peptides from APD	199 proteins predicted to be transmembrane	106 sequences from positive & negative training sets
Wang et al	BLASTP & Nearest-Neighbour Algorithm (NNA)	93.31%	25 composition & pseudo-amino acid composition features from initial set of 270 (for NNA)	870 peptides from CAMP (including some predicted)	8661 protein fragments randomly selected from UniProt	1136 predicted peptides from CAMP

By experimenting with all built-in kernels, we have found that a radial basis function with the default parameters performs best. The proposed SVM classification scheme takes an amino acid sequence as input and reports whether it exhibits antimicrobial properties or not, providing a probability estimate of the outcome.

Table 2 shows performance measures computed using a 10-fold cross-validation on the dataset described in the previous section. Accuracy is consistently high, however true negative rate is slightly lower than true positive rate, meaning the classifier will fail to recognize antimicrobial peptides slightly more often than non-antimicrobial ones. Still, all performance metrics score very well and made all the more credible by the high MCC values. Current classification schemes attained accuracies of 87.5%-93.2% for CAMP using 64 features [10], 90% for [25], [26] using an Artificial Neural Network (ANN) with 50 nodes (without cross-validation), 94% for [27] using an ANN with 44 descriptors and a validation set consisting exclusively of randomly synthesized peptides, 83.02% for [28] using 4 features and an SVM with a small dataset, and 93.3% for [29] using a combination of physicochemical properties and the Basic Local Alignment Search Tool (BLAST) alignment (with a sensitivity of ∼80.2%), as outlined in Table 1. Compared to the classification algorithms mentioned above, our classifier manages to achieve both high accuracy and high sensitivity and specificity while being trained on a diverse set (including both naturally occurring and artificial amino acid sequences) and employing a relatively low number of features.

**Table 2:** Maximum, minimum and average values of Accuracy, Sensitivity, Specificity and Matthews Correlation Coefficient (MCC) for a 10-fold cross-validation.

Values	Accuracy	Sensitivity	Specificity	MCC
Max	0.94	0.96	0.94	0.87
Min	0.89	0.92	0.87	0.78
Average	0.91	0.93	0.90	0.82

**Figure 1:**
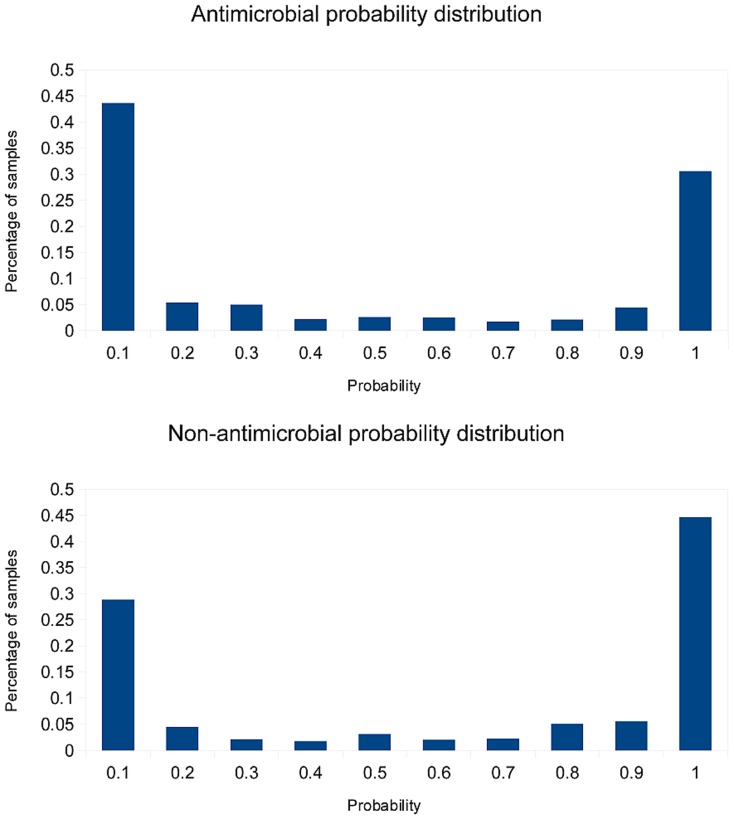
Distribution of predicted probabilities for antimicrobial (a) and non-antimicrobial (b) samples.

Figure 1 shows the probability distributions for antimicrobial/non-antimicrobial predictions for one of the cross-validation test sets consisting of ∼1050 peptides (results from the rest cross-validation runs are similar).

We selected PhytAMP, which contains experimentally validated plant antimicrobial peptides, in order to test our algorithm's predictive power against that of CAMP. We firstly used all the 271 antimicrobial peptides of PhytAMP (273 peptides except 2 that include non-standard amino acids). CAMP predicted correctly 252 out of 271 peptides (92.99%) whereas C-PAmP predicted correctly 270 out of 271 peptides (99.63%). Excluding from the initial PhytAMP dataset 59 peptides that had been included to our training set results in a subset of 212 peptides. When screening these 212 peptides, CAMP predicted correctly 197 out of 212 peptides (92.93%) whereas C-PAmP predicted correctly 211out of 212 peptides (99.53%).

### Scanning proteins for antimicrobial regions

We used our classifier to identify antimicrobial regions of length ranging from 5 to 100 amino acids [11] in all proteins of all plant species found in UniProtKB/Swiss-Prot. For each plant protein, all subsequences of a given length were tested using a sliding window (e.g. in order to find antimicrobial sequences of length 5, we tested subsequences spanning positions 1 to 5, 2 to 6, …, (*n*-4) to *n*, where n is the length of the protein). Sequences whose antimicrobial probability exceeds 90% have been stored in a database. These data have been created utilizing our Institution's (BRFAA) High Performance Computer Cluster consisting of 6 servers, each equipped with 2 x 6-core Xeon 2.66 GHz processors and 16 GB RAM.

### Database

**Figure 2:**
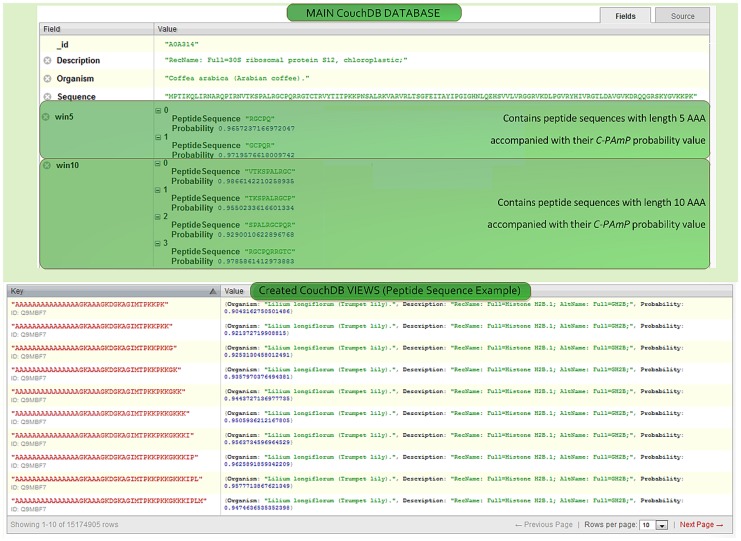
Snapshots from the C-PAmP Database.

As previously mentioned, we created a database in Apache CouchDB format (see Figure 2). Our database contains 15,174,905 antimicrobial sequences, whose probability of being antimicrobial is at least 90%. These sequences are derived from 33,877 proteins found in 2,112 plant species. It is worth noting that since proteins were scanned using a sliding window, many of these peptides overlap, or are subsets of one-another, so the number of unique subsequences of proteins is significantly lower.

Database records (documents) correspond to proteins, and each has the following fields:

- _id: contains the protein accession number (AC). For instance, “*A0A314*”.
- Description: Contains a brief description of the protein, extracted from UniProtKB/SwissProt. For instance, “*RecName: Full = 30S ribosomal protein S12, chloroplastic*”
- Organism: contains the name of the plant the specific protein belongs to. For instance, “*Coffea arabica* (arabian coffee)”
- Sequence: contains the sequence of the whole protein. For instance, “*MPTIKQLIRNARQPIRNVTKSPALRGCPQRRGTCTRVYTITPKKPNSALRKVARVRLTSGFEITAYIPGIGHNLQEHSVVLVRGGRVKDLPGVRYHIVRGTLDAVGVKDRQQGRSKYGVKKPK*”
- win5…win100, where applicable: contain peptides of corresponding length along with their respective C-PAmP antimicrobial probability. For instance, “*win*10”; “*Peptide Sequence: VTKSPALRGC*”; “*Probability*: 0.986”.

In addition, four CouchDB views were also created to speedup query processing time. In Figure 2, an example of view of the database with 10 out of 15,174,905 peptides is illustrated.

## Utility and Discussion

**Figure 3:**
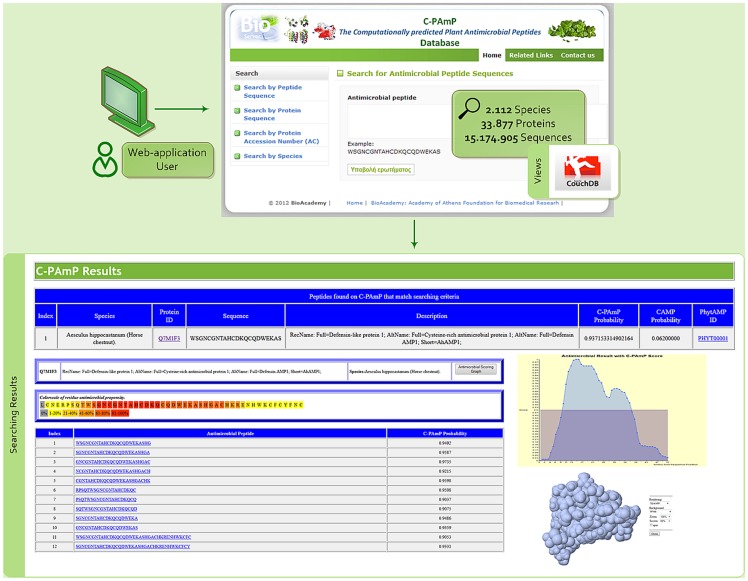
Snapshots from the web interface of C-PAmP Database.

This database is accessible via C-PAmP (see Figure 3), a web application that provides users with the ability to search and estimate the antimicrobial potential of individual peptides within a variety of plant proteins. Users can search by:

- Peptide Sequence: The application searches the database for proteins that contain the submitted peptide sequence and responds with the antimicrobial classifier score, also providing the CAMP antimicrobial score. In addition, if there is any experimental evidence for this peptide sequence either in PhytAMP database or in CAMP platform, then the proper links are provided to the user.
- Protein Sequence: The application searches whether the protein is contained in the database. If so, the corresponding species, protein name and AC, sequence and description are returned. Clicking on the protein AC displays the results of a search by protein AC (see below).
- Protein AC: The application returns some basic information on the requested protein along with information regarding the probability for antimicrobial action with respect to position within the protein (presented as both antimicrobial graph and heatmap). Moreover, a list of antimicrobial peptides derived from the protein is presented. Furthermore, the application presents the protein structure, if a corresponding Protein Data Bank (PDB) file is found. The antimicrobial graph and the corresponding heatmap show regions where antimicrobial peptide presence is prevalent: amino acids are coloured according to the number of antimicrobial peptides they are part of. The graph/heatmap value is the normalised weighted sum of C-PAmP probability scores of all the overlapping antimicrobial peptides.
- Species: The user selects the plant species of interest and the system retrieves the corresponding proteins with high-scoring antimicrobial peptides from the database.

**Figure 4:**
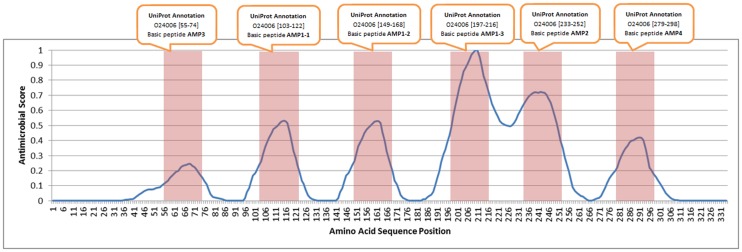
C-PAmP predictions for 6 antimicrobial regions in protein O24006 of Impatiens balsamina (Balsam) in comparison with the corresponding annotations in UniProtKB/Swiss-Prot.

Five examples using the C-PAmP tool are illustrated below. In the first example, according to [30] it is stated that in protein [Swiss-Prot:A4L7R7], plant *Pinus Sylvestris* (scots pine), Defensin-1 is found in the Chain from 34 to 83 amino acid with score 0.81 using CAMP, whereas no respective record in PhytAMP database was found. Using our approach, C-PAmP found that sequence to be antimicrobial with probability equal to 1. In the second case described in [31], antimicrobial activity (Defensin D1) was found in the whole chain of the protein [Swiss-Prot:P86972], plant *Nigella sativa* (black cumin), with CAMP scoring 0.79 and no respective record in PhytAMP. Again, C-PAmP classifies that protein as antimicrobial with 0.99 probability. In the third example Defensin-like protein 1 [Swiss-Prot:P0C8Y4] was found by [32] in plant *Dahlia merckii* (bedding dahlia). However, there is no relevant PhytAMP entry, whereas CAMP scores 0.91 and C-PAmP scores 0.99. In the fourth case, if we analyse protein [Swiss-Prot:O24006] of *Impatiens balsamina* (balsam), we see six peaks in its antimicrobial score graph. This finding is consistent with the corresponding annotations in Swiss-Prot as shown in Figure 4. Finally, the protein [Swiss-Prot: P01542] that is classified by the proposed prediction algorithm as antimicrobial with probability 0.98, is found in PhytAMP as antimicrobial too, whereas the specific peptide is absent in CAMP database.

In the presented examples we observe that C-PAmP provides information that is consistent either to experimental data from PhytAMP or to data retrieved from CAMP. The prediction scores for the presented antimicrobial peptides are higher in C-PAmP than in CAMP, providing stronger evidence in the correct direction. Therefore, we believe that C-PAmP can act complementary to the other two platforms, providing a comprehensive, large scale repository of strong candidate antimicrobial peptides found in plant species.

**Figure 5:**
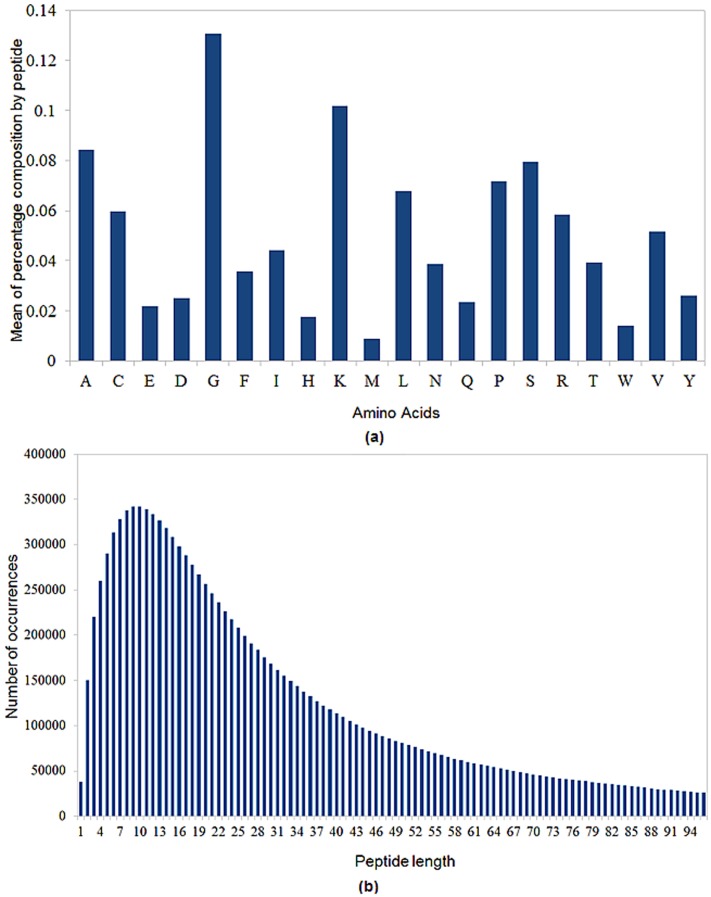
Statistics of the predicted plant antimicrobial peptides.

In Figures 5 (a) and (b) we can see some statistics concerning the computationally predicted plant antimicrobial peptides. According to them, the most probable peptide length is between 12 and 15 amino acids. In addition, the candidate antimicrobial peptides are found with high content of glycine residue, which may provide flexibility to the peptide structures. Peptides are also enriched in lysine, alanine, serine, proline, leucine and cysteine. They are poor in histidine, methionine and tryptophan, a trend also present in the PhytAMP peptides.

## Conclusions

C-PAmP is a database that contains computationally predicted antimicrobial peptides from plants. Peptides were selected by a highly performing classifier that tested all subsequences ranging from 5 to 100 amino acids of all proteins of all plant species in UniProt/SwissProt. The web interface of C-PAmP supports multiple types of queries and provides a lot of relevant information on peptides besides their probability score. C-PamP is the first database of its kind, offering such comprehensive information on predicted peptides and direct comparison with other predictors. It is the only database of predicted peptides dedicated to plants and the result of a large-scale computational experiment. C-PamP is driven by a powerful classifier, whose performance metrics on a diverse training set and classification results on PhytAMP lend credibility to its predictions. Since classification is based on a different approach than those of other studies and benefits from high performance, C-PamP can also provide an independent test to other, state-of-the-art antimicrobial activity predictors.

We hope researchers involved in novel drug design will use it to speed up the discovery component of antimicrobial peptide research.

### Availability and Requirements

C-PAmP is available at: http://bioserver-2.bioacademy.gr/Bioserver/C-PAmP/. Latest Sun Java (http://www.java.com) software should be installed in order to display protein structure PDB files associated with Protein Accession Number.
